# Europhen

**Published:** 1892-03

**Authors:** 


### EUROPHEN.

This new iodine product and antiseptic has now been on trial in the hands of the profession for several months. The results obtained by it have been generally favorable. It was designed to replace iodoform, and it was hoped also that it would prove a substitute for the time-honored specific medication in constitutional syphilis.

Europhen is a yellow, resinous powder, with an odor resembling that of saffron. It is insoluble in water and glycerine, but dissolves easily in alcohol, ether or chloroform. Solutions decompose rapidly. Europhen is five times lighter than iodoform.

Clinically it appears to be a perfect substitute for iodoform. It prevents the development of micro-organisms, and it is soluble in the same menstrua as iodoform. It has the advantage over iodoform that large percentages of it will remain in solution in fixed oils. Furthermore, it is relatively free from odor and non-toxic. Concerning the specific use of europhen, Dr. Eichhoff publishes his experience in a number of cases. (*Ther. Monat.*)

"These were favorably influenced by it. Ulcus molle healed quickly in two cases by means of the simple dusting on of europhen."

"Constitutional syphilis in all stages reacted to europhen by local use, as well as in the form of subcutaneous injections.

"From the clinical reports, it is found that five cases of hard chancre were completely healed after treatment, averaging fourteen days, by the mere dusting on of europhen.

"Four cases of large condylomata were treated with like results.

"But europhen has an extraordinarily favorable effect in syphilitic affections, not only as locally used, but, according to Dr. Eichhoff, *the constitutional symptoms are caused to disappear by means of subcutaneous injections of this remedy.* The injections are perfectly painless and show no appearances of reaction, either local or general.

"If we use, hypodermically, rather large doses of europhen, *e. g.*, 1½ grains, without having begun first with smaller doses, many patients complain, after the first injections, of headache and abdominal pains. The later injections, on the contrary, are, without exception, well borne.

"It appears that the patient must become accustomed to the remedy. Dr. Eichhoff treated seven cases of constitutional syphilis in various stages with single, daily injections of europhen oil, and all were discharged, as free from all signs of syphilis, in a relatively short time."

Non-venereal skin affections were not influenced by europhen.

It appears that europhen is a useful addition to our pharmacopœia, and it is hoped that the favorable reports as to its value will be sustained by further trial and experience.

